# Training Traditional Birth Attendants on the Use of Misoprostol and a Blood Measurement Tool to Prevent Postpartum Haemorrhage: Lessons Learnt from Bangladesh

**Published:** 2014-03

**Authors:** Suzanne Bell, Paige Passano, Daniel D. Bohl, Arshadul Islam, Ndola Prata

**Affiliations:** ^1^Bixby Center for Population, Health and Sustainability, School of Public Health, University of California at Berkeley, CA 94720-7360, USA; ^2^RDRS Bangladesh, Uttara, Dhaka1230, Bangladesh

**Keywords:** Blood measurement tool, Misoprostol, Postpartum haemorrhage, Qualitative, Traditional birth attendant, Training, Bangladesh

## Abstract

A consensus emerged in the late 1990s among leaders in global maternal health that traditional birth attendants (TBAs) should no longer be trained in delivery skills and should instead be trained as promoters of facility-based care. Many TBAs continue to be trained in places where home deliveries are the norm and the potential impacts of this training are important to understand. The primary objective of this study was to gain a more nuanced understanding of the full impact of training TBAs to use misoprostol and a blood measurement tool (mat) for the prevention of postpartum haemorrhage (PPH) at home deliveries through the perspective of those involved in the project. This qualitative study, conducted between July 2009 and July 2010 in Bangladesh, was nested within larger operations research, testing the feasibility and acceptability of scaling up community-based provision of misoprostol and a blood measurement tool for prevention of PPH. A total of 87 in-depth interviews (IDIs) were conducted with TBAs, community health workers (CHWs), managers, and government-employed family welfare visitors (FWVs) at three time points during the study. Computer-assisted thematic data analysis was conducted using ATLAS.ti (version 5.2). Four primary themes emerged during the data analysis, which all highlight changes that occurred following the training. The first theme describes the perceived direct changes linked to the two new interventions. The following three themes describe the indirect changes that interviewees perceived: strengthened linkages between TBAs and the formal healthcare system; strengthened linkages between TBAs and the communities they serve; and improved quality of services/service utilization. The data indicate that training TBAs and CHW supervisors resulted in perceived broader and more nuanced changes than simply improvements in TBAs’ knowledge, attitudes, and practices. Acknowledgeing TBAs’ important role in the community and in home deliveries and integrating them into the formal healthcare system has the potential to result in changes similar to those seen in this study.

## INTRODUCTION

The World Health Organization (WHO) defines a traditional birth attendant (TBA) as “a person who assists the mother during childbirth and initially acquired her skills by delivering babies herself or through an apprenticeship to other TBAs” (1). Beginning in the 1970s, the WHO advocated for the training of TBAs as a strategy to reducing the maternal and neonatal mortality and morbidity occurring in low-resource settings during home deliveries. However, in the 30 years following the WHO's recommendation, the majority of reviews on the impact of TBA training did not find compelling evidence to promote it as a strategy to reducing maternal mortality (2).

In light of these studies, a consensus emerged in the late 1990s among leaders in global maternal health that TBAs should no longer be trained in delivery skills and should instead be incorporated into the skilled birth attendant strategy as promoters of facility-based care (3). The safe motherhood initiative began advocating for skilled birth attendants (SBAs) at every birth and increased access to emergency obstetric care (EmOC) in low-resource settings. Unlike most TBAs, the SBAs receive formal medical training and are better equipped to manage complications. However, since inequities in the distribution of SBAs are pervasive and resources to train them are limited in many developing countries, these goals remained unattainable (4). Worldwide, 34% of births still occur without an SBA present (5). To mitigate the effects of the shortage of healthcare workforce, many low-resource countries have continued to invest in the training of TBAs and other cadres who practise in their own communities. Appropriate technologies, for example misoprostol to prevent PPH and blood loss measurement tools to measure blood loss, create new opportunities for re-evaluating the role of TBAs at delivery.

In Bangladesh, more than 70% of women deliver at home and only 32% of births are attended by a medically-trained provider (6). Bangladesh has an extensive rural health infrastructure but the challenge of making SBAs available to all women who need them has been insurmountable to date (7). Acknowledgeing that most women are still choosing to deliver at home, the Government launched a programme to train two additional cadres of community health workers to be SBAs. However, implementation was challenging, and in the five years following the community SBA initiative, the new cadres had conducted only 0.1% of all deliveries (8). Rates of skilled attendance at birth have steadily increased over the last decade but with a very low baseline, the complete transition to facility-based delivery will take decades (Figure) (6,8); the majority of births (63%) are still attended by TBAs, trained or untrained (6).

Qualitative research has demonstrated that some women prefer being assisted by a TBA compared to an SBA, particularly if the SBA is young, unmarried, and without children (9). Because TBAs live and work within the social and cultural matrix of the community, they often understand women's needs and are better positioned to influence women to choose safer options in pregnancy (9,10). In addition to advice and information, TBAs are often well-situated to coordinate referrals to health facilities (10). Previous work has shown that incorporating TBAs into the formal healthcare system can increase skilled birth attendance and utilization of services (11). Additionally, researchers working in Zimbabwe determined that TBAs serve as the bridge between biomedical and traditional medicine, providing women with greater re-assurance of positive outcomes than what is available to them in standard antenatal care (ANC) (12). With better coordination, TBA integration could leverage TBAs’ unique position within the community to link women with the formal healthcare system (11). Merely training TBAs in safe motherhood interventions without the necessary technologies, infrastructure, and support cannot make TBAs effective (12-15). After decades of safe motherhood programming, it has become apparent that isolated interventions are not sufficient to reduce maternal mortality.

Only a few high-quality studies have demonstrated TBAs’ contribution to significant reductions in the incidence of postpartum haemorrhage (PPH), the leading cause of maternal death worldwide (16-18). In Gambia, qualitative findings demonstrated that TBAs have the potential to contribute to the management of PPH in many cases but poor integration, weak infrastructure, and delays in referral inhibited TBAs’ impact (19). One way TBAs could make an impact on reducing PPH is through the use of misoprostol, which was recently included in the WHO's Essential Medicines List after a review of the existing evidence (20). The WHO now supports administration of misoprostol by non-skilled birth attendants at home deliveries as well (21). Bangladesh also recently approved community-based provision of misoprostol for the prevention of PPH.

**Figure.**
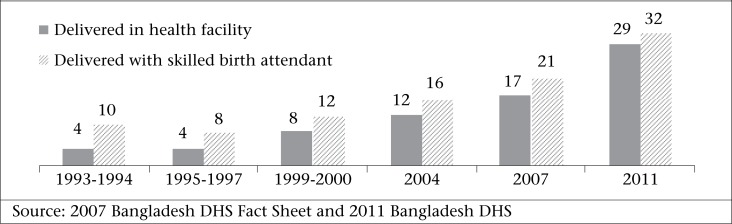
Percentage of deliveries in a health facility and skilled birth attendance in Bangladesh, 1993-2011

This study strove to qualitatively assess the experiences of health workers after a TBA training on the use of misoprostol and an absorbent blood measurement tool. The primary objective of this study was to gain a more nuanced understanding of the full impact of training TBAs to use these tools for the prevention of PPH at home deliveries. We present quotes from interviews with TBAs, community health workers (CHWs), and managers that represent the main perceived changes relating to knowledge, service delivery, community relations, and interaction with the formal healthcare system following the TBA training. If integration of TBAs becomes a safe motherhood priority, our data indicate that TBAs could serve as the bridge between the formal healthcare system and the rural communities with otherwise limited access to it.

### Context of the study

This qualitative study, conducted between July 2009 and July 2010, was nested within larger operations research testing the feasibility and acceptability of scaling up community-based provision of 600 µg of misoprostol and a blood measurement tool for the prevention of PPH. The blood measurement tool is a locally-produced device that is placed under the woman after delivery of the baby that can capture blood loss up to 500 mL, which is the threshold for PPH. The larger study enrolled 118,500 women from six northern rural districts of the Rangpur division in Bangladesh and tracked the experiences of 77,337 women who delivered during the study period. The pregnant women involved in the study were the clients of a reproductive health programme implemented by a well-established, local non-governmental organization (NGO)—Rangpur Dinajpur Rural Services (RDRS). The project was led by icddr,b in Dhaka in collaboration with the Bixby Center for Population, Health and Sustainability at the University of California, Berkeley and Venture Strategies Innovations (VSI).

RDRS conducts ANC in 203 clinics across the six study districts and trains and oversees approximately 600 TBAs who are affiliated with these clinics. RDRS's 62 trained CHWs supervise these TBAs, although their field supervisory capacity (in terms of labour and delivery) is limited as they themselves are not trained in midwifery skills. TBAs and CHWs of RDRS work collaboratively: first, the TBAs recruit clients from the communities to register with RDRS, after which these women can receive ANC from CHWs at the clinics, purchase a clean delivery-kit (CDK) for a nominal fee, and choose to be assisted during home deliveries by one of RDRS's TBAs. ANC provided by RDRS's clinic-based CHWs includes measurement of weight, height, and blood pressure, urine testing, iron and folic acid tablets, physical examinations of the mother, regular assessment of the baby's position and vitality, group information sessions, and referrals for ultrasound or other tests. TBAs typically spend one day per week in the ANC clinic assisting their CHW supervisors.

In total, 696 field staff, 588 of which were TBAs of RDRS, received two days of training on misoprostol and the use of the blood measurement tool (mat) in the spring of 2009, after which these two new technologies were added to RDRS's existing CDKs at no extra charge to clients. The training covered various aspects of misoprostol (function, dosage, timing of administration, side-effects and their management, etc.) and the use of the blood measurement tool which was designed to indicate the onset of PPH. It also covered information on identifying high-risk pregnancies, danger signs in pregnancy, referral procedures, stages of labour, resuscitation of the newborn, maternal infection, and general use of the new CDKs. Trainers focused their instructions on PPH and other maternal complications requiring referral. Monitoring of TBAs occurred through January 2011. Results from an evaluation of the TBA training are published elsewhere (22). Ethical approval for the study was provided by the Committee for the Protection of Human Subjects at the University of California, Berkeley (CPHS # 2010-01-619).

## MATERIALS AND METHODS

In total, 87 in-depth interviews (IDIs) were conducted at three time points during the study: July 2009, two months after implementation (T1); February 2010, 7 months after implementation (T2); and July 2010, 13 months after implementation (T3). The study team conducted interviews with 63 TBAs, 12 CHWs, 8 managers of RDRS, and 4 government-employed family welfare visitors (FWVs) who shared clinic space with RDRS's CHWs. The FWVs are trained to conduct pre- and postnatal care, provide family planning, and conduct deliveries. The purpose of interviewing at different time points was to determine how TBAs’ practices and perception of the communities’ reaction to the intervention changed over time. After reading the transcripts following the second round of interviews, we felt we reached saturation with the TBAs and sought additional viewpoints from CHWs and managers.

TBAs were the primary study population since they were the direct users of the new technologies and they had the most extensive contact with the communities served. To gain a wider range of perspectives across RDRS's hierarchy, CHWs and managers were interviewed to triangulate and validate reports by TBAs and to include their perspectives on the changing relationships within the organization and within the communities. We included a small number of FWVs to better understand the opinions of government employees about the intervention but FWVs were not included in this analysis because the focus of their interviews diverted from the objective of this paper.

We conducted IDIs as a method to maximize diversity and honesty in responses. Interviewees were purposefully selected from each of the six study districts to represent the geographical diversity both within each district and across the region. We also conducted three focus group discussions (FGDs) during T2 (two groups with TBAs and one with CHWs) to assess social norms within RDRS and to determine whether group norms differed from individual reports, which they did not. The FGDs with TBAs involved eight TBAs each, and the FGD with CHWs involved nine CHWs; there was no overlap between in-depth interviewees and FGD participants. All subjects provided informed consent prior to participating in the interviews or FGDs.

Researchers developed three IDI guides, each corresponding to one of the three time periods in which interviews were conducted. All interviews and FGDs were semi-structured and exploratory in nature. Topics covered included knowledge gained from the training, changes in practices and workload, perceptions of the communities’ reaction to the interventions, and changes in relationships since the training. Interviews were conducted in Bangla, recorded by study personnel on a digital recorder, and transcribed and translated from Bangla into English by an independent contractor not affiliated with the study.

Two researchers conducted computer-assisted thematic data analysis using ATLAS.ti (version 5.2) (23). Researchers created an initial codebook, derived inductively after their first reading of the interviews. After dividing all primary documents and coding the first half of the interviews, they collaboratively revised their codebook and added or merged codes based on findings. They completed the coding process with the latter version of the codebook. One check of inter-coder reliability was done at the onset of the coding; results were satisfactory, and differences were not deemed an impediment to the analysis.

After coding all primary documents, researchers collaboratively combined codes into overarching themes. The goal of the analysis was to inductively capture cross-cutting themes relating to changes resulting from the training that emerged throughout all primary documents; we did not analyze interviews/FGDs by time period or by group of interviewees. The themes we discovered present a broad range of interviewee-reported changes that arose as a result of the TBA training.

## RESULTS

All TBAs and CHWs interviewed were female; five of the eight managers interviewed were male. The average length of the IDIs was approximately 36 minutes, and the average length of the FGDs was 48 minutes.

Four primary themes emerged during the data analysis, which all highlight changes that occurred following the TBA training on misoprostol and the blood measurement tool. Theme One describes the interviewee-perceived direct changes linked to the two new interventions. These are changes that are a direct result of information or instruction provided during the TBA training that were anticipated by the investigators (e.g. use of misoprostol and the mat at home deliveries, decrease in the amount of blood loss following the use of misoprostol, etc.). The following three themes describe the indirect changes that occurred. Theme Two illustrates the perceived strengthened linkages between TBAs and the formal healthcare system. Following the training, respect for TBAs and the TBAs’ new knowledge improved the relationship between TBAs and CHWs and enhanced collaboration between TBAs and facility-based providers. Theme Three presents the interviewee-perceived strengthened linkages between TBAs and the communities they serve. In order to explain the new technologies, TBAs spent more time interacting with women and their families as the value placed on these new services increased in the eyes of the communities. Theme Four captures the interviewee-perceived improvement in service utilization and quality of services. After the TBA training, the TBAs provided new and improved services to their respective communities. All interviewees had the experience that, as information about misoprostol and the blood measurement tool spread, increasing numbers of families became interested in registering for ANC. Below, we discuss each theme in detail, incorporating illustrative quotes from the data.

### Theme 1: Direct intervention impacts

The first theme captures the most frequently-identified interviewee-perceived direct impacts of the training intervention. These include, but are not limited to, safer home delivery, less blood loss, less need for referral, improved blood loss measurement, better knowledge of when to refer, timely placental expulsion, and decreased maternal mortality. According to all TBAs interviewed, their knowledge increased immediately after the training. One of the trainees recounts what she learned in the training:

How to motivate mothers, bring them to the hospital, what should be done during delivery, how to use the bag. After delivery, check whether mother has another baby or not, then give mother three tablets. If only one child, [whether] placenta come out or not, give the tablets to the mother. Mother will have less bleeding. Uterus will be in proper size. They have given a paper; after delivery, mother will sit on the paper, then they check the amount of blood loss. If small area of the paper is soaked with blood, it is normal, and if fully soaked with blood, then it is abnormal: mother is having excessive bleeding, she has to go to a doctor (TBA, T1).

This knowledge was subsequently passed on to the mothers in their respective communities:

Before, village mother did not know and understand many things. Using tablet and mat, mothers are aware now. Mothers know the benefit of mat and the tablet (CHW, T3).

Within months of using the two new interventions, our data indicate that RDRS staff members were highly impressed with the power of the two latest additions to their CDK. As one manager succinctly stated:

Trained TBAs, misoprostol tablets, and mat—these three are the instrument[s] of safe delivery (Manager, T3).

Many TBAs shared how misoprostol saves women's lives by preventing the need for transfer to a facility:

Before, this tablet was not available. Mothers had retained placenta and were taken to the hospital for excessive bleeding. Now, taking misoprostol tablet, mothers do not have retained placenta and [there is] no need to go to the hospital. This tablet saves the life of mothers (TBA, T2).

It is clear from this quote and others that TBAs understood how misoprostol works and how it can make delivery safer by decreasing placental retention and blood loss, thus reducing the need for transfer. An RDRS manager reiterated the importance of averting complications requiring referral, stating:

If you want to refer a woman from a remote area, it takes time. By that time, the woman is actually dead already (Manager, T3).

Virtually all trained TBAs demonstrated a clear understanding of how to use the new blood measurement tool and could describe at which cutoff point the mat signals a need for immediate transfer to the hospital:

Before, I didn't know the amount of blood loss. Blood came out on the plastic [but] I didn't understand the amount. Now the mat indicates the amount of blood loss. If the mat is fully soaked with blood, [it] is excessive bleeding. That mother should not be kept at home, the mother should be taken to the hospital (TBA, T1).

Additionally, many interviewees spontaneously drew a connection between the benefit of the two new interventions and a reduction in maternal deaths:

Before, there were no misoprostol tablets. Many mothers had heavy bleeding, placental tears, and pieces remained inside the mother. We had no prevention measure. The mothers who stay at far distance died on the way while taking to hospital. Now, we have the tablet; many mothers [are] safe from death (CHW, FGD, T2).

TBAs also commented on the vast improvements they have witnessed since they began their careers, emphasizing that they have finally gained access to effective tools:

Before, mothers faced many troubles—the placenta didn't come out easily. Village TBAs inserted their hands and gave pain to the mother. During delivery, many untrained TBAs hurt the mother's vaginal space. Later, mothers suffered from disease. Now, these [things] do not happen (TBA, T2).

These quotes represent the direct and immediate impact that RDRS staff experienced as a result of the implementation of these two interventions. The changes that occurred were the intended result of the training, and the interviewees’ responses indicate that the TBAs understood this intent. The combination of the TBAs and the intervention tools was essential; the TBAs were the primary vehicle of the intervention; yet, it was the modern technology that directly elicited changes in the safety in home deliveries. The direct impacts of these interventions, as described by interviewees, could not have occurred without the training.

### Theme 2: Strengthened linkages between TBAs and the formal healthcare system

One key indirect outcome of the intervention was the development of stronger linkages between TBAs and CHWs. Because of the intervention, demand from researchers for monitoring data increased, which required CHWs to obtain accurate information about each RDRS-registered woman who delivered in the community. This led to increased interaction between CHWs and TBAs who actually collected the delivery-related data from mothers. One TBA reported on the change in the level of communication:

The CHWs stay in the clinic, and I visit house to house. Now, much [more] communication happens between both of us due to the tablet and the mat (TBA, T1).

Since most TBAs have received little to no formal education, distinct social divisions exist between TBAs and CHWs. However, RDRS's joint training and partner assignments set an expectation of mutual respect. The high school-educated CHWs quickly realized the need to respect their TBA partners in order to maintain good relations and to facilitate accurate data collection:

If we do not behave well with the TBAs, they will not bring correct information about the mothers (CHW, T3).

Through this strengthened partnership, TBAs solidified their ability to give accurate advice, and CHWs increased their awareness of ground realities for community women. One CHW expressed admiration and respect for the work of the TBAs she supervises:

They know many things which I do not know. Going to the field with TBAs, I see and learn many things (CHW, T3).

Another benefit of the intervention was stronger linkages forged between TBAs and medical professionals in referral hospitals. Once TBAs learned how to use misoprostol and the mat, their abilities were immediately improved. Access to misoprostol and the blood measurement tool gave them the ability to prevent and manage PPH, the deadliest threat to women in childbirth worldwide. The ability to use these simple tools, backed by training in recognition of danger signs, earned them respect not only from communities but also from skilled professionals. Most importantly, their active referral behaviour led to stronger relationships with facility-based providers working in referral hospitals. One TBA remarked:

We received training from RDRS. Village people give importance to trained TBAs. Hospital doctors also give importance to our work. We always take patients to the hospital. We know the doctors of the hospital very well (TBA, T2).

Learning about best practices in supervision was another important gain that strengthened the ties between TBAs and their supervisors. By implementing the study in collaboration with icddr,b, RDRS staff learned how to be more systematic in monitoring and evaluation. One RDRS manager remarked on the value added:

What we have learnt from this study [is] how supervision is done actually. Our supervisors learned from them [icddr,b staff]. They have supervision checklists, they are used to visiting the houses…Some of our supervisors were involved with icddr,b in the district level. That was the positive side for us, [learning] how to do supervision and monitoring in the field at every level (Manager,T3).

Before TBAs had access to misoprostol, they relied primarily on village doctors (who practise allopathic medicines) if serious complications occurred during home deliveries, such as retained placenta or PPH. TBAs told numerous stories about their village doctor partners, which revealed risky techniques (e.g. injections to induce labour) and considerable delays in situations where immediate referral appeared to have been necessary:

First, when pieces of cloth were fully covered with blood, we changed and gave another piece of cloth. When that was also covered with blood, we called the [village] doctor. When village doctors were helpless, then we sent the mother to the hospital. Now, getting the mat, I myself send the mother to the hospital. While going to the hospital, I take the soaked mat with me; otherwise, how doctor will realize the amount of blood loss? (TBA, T2).

An unexpected outcome of the intervention was that the new knowledge, effective tools, and professional contacts that TBAs obtained meant they no longer felt the need to utilize the services of village doctors. This appears to have substantially reduced both the first and the second delays (recognition of maternal complications and arrival at a health facility). Not a single TBA mentioned regret over the dissolution of their prior partnerships, and CHWs and managers were categorical in their assertion that the elimination of the need to call village doctors was a positive outcome of the training.

### Theme 3: Enhanced communication and trust between TBAs and their communities

The third theme addresses the strength of the relationship between TBAs and the communities they serve, a critical factor in the success of this intervention. Our data offer insight into the perceived changes in the quantity and quality of interactions between providers and clients and the changes in TBAs’ status that occurred following the training on misoprostol and the mat.

When the study began, a careful system of postpartum monitoring to capture actual use of misoprostol was designed. Immediately after a registered woman's delivery—whether an RDRS's TBA was present at the delivery or not—an RDRS's TBA had to visit the woman during her postpartum period to check her condition and collect the empty (or unused) misoprostol packets. Postpartum visits were already a part of RDRS's protocol but the emphasis on collecting information relating to delivery and the use of misoprostol and the mat was new. Although TBAs faced a heavier workload, this was taken in stride by most. The interviewees’ perception was that communities responded positively to the additional attention from TBAs, leading to a greater connection between TBAs and families:

When misoprostol activities [were] tacked [onto] our programme, our volume of work has increased. Now, [we] are going to the mother more frequently. We are keeping mothers in close follow-up to know whether they are taking the tablet or not, and to know the side-effects, we are doing more counselling to the mother, and we are explaining more to the mother about the use of misoprostol tablet, [the] mat, and the delivery-kit (Manager, T3).

An unexpected perceived shift in TBAs’ status also increased contact between TBAs and the communities. Reflecting back on her social status in the beginning of her career, one TBA explained:

When we started working, mothers never listened to us, they didn't give [us] permission to enter their house. They would say, “Why did you come, what do you want? Whether mother will go with you or not, we will decide that.” Now nobody does that (TBA, T2).

Since the launching of the new interventions, TBAs reported a distinct change in how they are received by clients. They reported being approached and invited into houses, being offered special dishes, having their advice adhered to carefully, and receiving gifts of appreciation following births. One TBA stated:

That time people did not like to talk with me. After the availability of the mat and the tablet, my importance [has] increased. Now, people respect [me] much and talk with me (TBA, T2).

A CHW added:

Before, TBAs talked less but now they explain clearly about tablets and the mat. For this reason, they talk more with mother…Now, everybody relies upon TBAs. People respect the TBAs much [more] than before (CHW, T3).

One possible explanation for the increase in registration of RDRS numbers for ANC, as reported by interviewees, is that women are being increasingly ‘allowed’ to access maternal health services, which may be a result of increasing awareness among decision-makers in rural communities. One TBA observed:

Male persons come with the women and listen to us. After listening, they say these are all good and important words (TBA, T2).

Furthermore, confidence in their improved maternal health knowledge, paired with greater respect from colleagues and communities, appears to have enabled the TBAs to take a more authoritative stand with community members when it comes to protecting women's lives:

When mothers have some complications and we tell them to go to a doctor, sometimes some people say to us, “What kind of a doctor are you? You are unable to identify the problem and [are] telling us to go to another doctor.” We say, “Everything [is] not possible by us. There are more efficient doctors than us, [and] for your benefit, I am telling you to take the mother to an experienced doctor” (TBA, T2).

In addition to TBAs’ confidence in asserting their opinions, the interviews revealed that TBAs’ advice is now more readily accepted. A critical component of TBAs’ ability to refer is whether the family adheres to the TBAs’ advice. Most TBAs reported a noticeable reduction in resistance to hospital referral:

People listen to us and rely on us much [more] than before. If we tell them to go to the hospital, they follow that. Before, they used to request us to try again to do the delivery at home. Now, they take the mother to the hospital. They take me with them (TBA, T2).

TBAs did report having to spend extra time and energy explaining the new interventions to mothers-in-law, and occasionally husbands, who hold decision-making power in the family. As one TBA explained:

Some mothers-in-law ask me, “Before, people didn't use this tablet; so, why is this required now?” I say these are a new arrival. After delivery, mother should not have heavy bleeding. For that reason, this tablet should be given to the mother (TBA, T2).

Interestingly, there was much less resistance to the new interventions reported from husbands compared to that encountered from the elders in the families.

Overall, the additional time spent with pregnant women and their families seems to have improved relations between TBAs and their clients, resulting in more business and greater economic stability for TBAs. One TBA stated:

As much mothers will be healthy, my name will spread everywhere (TBA, T1).

This TBA's remark illustrates the link between positive birth outcomes, pleased clients, and an increase in demand for TBA services.

### Theme 4: Increased service utilization and quality of care

Improvements in overall service quality and utilization were not direct aims of the training but the interviewees’ perception was that community attitudes towards ANC, registration rates in ANC, and demand for trained birth attendants improved following the training. According to the interviewees, as TBAs’ knowledge about safe motherhood expanded, this information appears to have been directly passed on to the communities they served, altering the people in the communities’ perceptions about the value of RDRS services. In describing recent changes in women's attitudes towards ANC, a TBA observed:

Before, people didn't give much importance. Now, getting misoprostol tablet, people are much [more] aware and give much importance. People say, “Giving 15 taka, we get treatment up to nine months. We get the delivery bag and a trained TBA for delivery” (TBA, T2).

This statement highlights an increased awareness about the importance of preventative care in pregnancy, labour, and delivery. In line with this increased desire for ANC services, both CHWs and TBAs described a perceived increase in registration rates in ANC after the new interventions were introduced:

Now, registration numbers are high due to the tablet and the mat. Now, everybody knows mothers die due to heavy bleeding. Our TBAs give this information to the mother when they visit their house. When mothers understand that the tablet will help them for having less bleeding, they feel interested and come to the clinic for the bag (CHW, T3).

This sudden influx of women registering for ANC increased both TBAs’ and CHWs’ workload, as mentioned in many interviews.

Most TBAs reported that the number of deliveries they conduct monthly increased substantially following the launch of the new CDK but interviewees rarely expressed negative attitudes about this change:

Workload increased. This is good because people are getting benefit…. Before, very few people took the bag. Now, [because of the] tablets, everybody is interested in taking the bag (CHW, T3).

The quality of TBAs’ work also appears to have increased. Many managers and CHWs noted distinct improvements following the training:

TBAs say now they feel much interest in doing their work. Now [there is] much demand for TBAs in the village. Now, TBAs [are doing many] deliveries. TBAs say now they are doing much more effective work. Before, TBAs didn't understand mothers’ excessive bleeding. Using the mat, the amount of blood loss is measurable. If the mat is fully soaked with blood, the mother is taken to the hospital. The mother's life is safe (CHW, T3).

Changes in the quality of work were not limited to TBAs. Upazila (subdistrict) meetings played a key role in increasing staff communication and accountability at all levels while encouraging a climate of learning and continuous quality improvement in services. An RDRS manager explains:

Besides basic training, TBAs used to come to the upazila for their honorarium. On that day, supervisors and CHWs of that upazila are present. Every TBA has to present how many deliveries she has done that month. If there is any difficulty or complications, they share and discuss so that, in the future they can correct that. They used to take their supplies from there and their honorarium. It was like refresher training (Manager, T3).

Overall, the unanticipated perceived changes in service utilization and quality of care were welcome as women's attraction towards services and enhanced work performance among the staff can only increase the proportion of women having safer deliveries.

## DISCUSSION

To our knowledge, this is the only qualitative study that has investigated perceived community-wide changes associated with the training of TBAs on the use of misoprostol and a blood measurement tool (mat). Existing qualitative literature focuses primarily on knowledge, attitudes, and practices as they relate to TBAs’ training (19,24) or on the role TBAs play within their communities (12,13,25). Our data clearly demonstrate that new technologies create new opportunities for TBAs and that, from the perspective of interviewees, 600 µg of oral misoprostol and a blood measurement tool are acceptable and in high demand in the study area. The results show that training TBAs on the use of these devices can facilitate a wide range of positive changes for pregnant women, communities, maternal health workers, and non-governmental organizations (NGOs).

The interviewees’ perceptions are that training TBAs and their CHW supervisors resulted in broader and more nuanced changes than simply an improvement in TBAs’ knowledge, attitudes, and practices. Following the training, a number of impacts directly resulted from the knowledge gained through the training and from the rapid uptake of the two interventions. In addition, a number of indirect impacts emerged from the qualitative interviews. After receiving the training, interviews indicate that TBAs became more respected within the formal healthcare system. Second, they better understood the importance of immediate referral, appearing more inclined to directly refer women to a health facility, if needed, instead of using the services of village doctors with dubious qualifications. Communication and trust between TBAs and community members increased, according to the interviewees, as the TBAs’ knowledge and status improved. With the addition of these two new interventions and increasing communication between TBAs and communities, the quality of care and the demand for RDRS services also increased. In general, the interviewees feel the intervention enhanced TBAs’ ability to conduct safer deliveries and link pregnant women with the formal healthcare system when needed.

The range of positive effects that interviewees reported occurring following the TBA training was unexpected and may have been unique to the specific context. The cohort of TBAs described in this study was linked to the formal healthcare system (referring for ANC and EmOC) even before the intervention took place as evidenced in three ways. First, they actively promoted ANC, which later included promotion of misoprostol and the mat. Second, the TBAs were trained to refer any pregnant women with conditions that might make delivery risky (e.g. malpresentation, etc.). Rather than trying to keep the clients for themselves, TBAs avoided risk by strongly encouraging any women with antenatal risk factors to deliver in a hospital. We hypothesize that this willingness is partly attributable to the fact that RDRS pays TBAs a monthly honorarium, which reduced their economic insecurity and their dependency on clients’ donations of money and/or gifts following delivery. Third, our data (not presented here) also indicate that TBAs understand the risks associated with delivery and the postpartum period. Since no TBA wants to be blamed by the communities she serves for problems that may occur in a complicated delivery, they were watchful and adamant about referral at the first sign of complications. Our data support previous observations that rural Bangladeshi TBAs strive to be accountable to the communities that they serve because their reputation directly impacts their livelihood (26). These three factors make this group of TBAs and the TBA training unique and also present a model to be replicated in other settings. Acknowledgeing TBAs’ important role in the community during home deliveries and integrating them into the formal healthcare system has the potential to result in changes similar to those seen in this study.

The ultimate lesson learnt is that, other than evaluating knowledge acquisition and retention in relation to the TBA training, programmes should also be aware of possible changes taking place in other aspects of service delivery and in the community. We were only able to capture these perceived changes through the perspective of those involved in the project but we feel the consistency with which these changes were cited throughout the interviews indicates that something real was happening in the community and in TBAs’ relationships following this training. The TBA training used evidence-based technology that is being incorporated into maternal and child health programmes throughout the developing world but it took place within a system where TBAs are valued and where they operate within a larger organization instead of independently. We believe the context of support and respect is as important as the effective technologies, and it explains the wide range of positive perceived changes in terms of TBA and community knowledge, communication, demand for safer services, and quality of care. Similar changes could be anticipated in other settings with a similar TBA culture where TBAs are integrated into the formal healthcare system. Quantitatively capturing the externalities of a similar TBA training would be interesting and is an important future area of study.

### Limitations

Our study has a few limitations. All data were based on self-report, and any of the interviewees might have altered their responses in an effort to please their interviewer or overstated the impact of the interventions to reflect positively on the programme. We hope that the number of interviews and the number of interviewee groups reduced the chances that these misleading responses would occur regularly enough to emerge as a theme in the data. In addition, these providers were not paid for their participation in the project, which, as findings indicate, actually increased their workload. Thus, interviewees had no real incentive to portray the programme as a success.

In addition, this intervention was laid on top of RDRS's well-established maternal health programme in a country with a long history of community-based healthcare delivery; thus, the reception of the programme by the community may be specific to the context of the study. Certain programmatic features may have reinforced the impact of the intervention. For example, the fact that TBAs work once a week in the clinics alongside CHWs could have served several strategic purposes: it could have strengthened interpersonal relations between CHWs and TBAs as the new interventions were being introduced; it could have continuously refreshed TBAs’ knowledge about how to use the new technologies; and it could have enhanced TBAs’ credibility as health providers in the eyes of the community through their association with the RDRS clinic. In addition, we used non-probabilistic sampling. The generalizability of our findings may be limited as a result of these factors. Our results provide insights into how TBAs and other RDRS health workers experienced this intervention and what they perceived some of the effects to be but the data alone do not definitively demonstrate that this intervention would elicit these changes in other settings.

### Conclusions

This study illustrates that, from the perspective of those involved in the study, a number of positive and unforeseeable changes came about as a result of the training of birth attendants. This more nuanced understanding of the impact of training demonstrates the potential impact TBAs can have when equipped with lifesaving technologies and information and are integrated into a formal healthcare system. Incorporating misoprostol and the mat into the CDK appears to have made it easier for TBAs to perform the aforementioned three key roles: promote registration for ANC, encourage hospital delivery for women with higher-risk pregnancies, and make emergency referrals during delivery, as needed. According to the interviewees, the training increased communities’ respect for the TBAs, improving the likelihood of adherence to TBAs’ advice, especially with regard to hospital referral. The new technologies also stimulated community interest in other maternal health services as reported in the interviews. For example, interviewees cited an increased interest in ANC registration, subsequently increasing the number of births protected from postpartum haemorrhage by administration of misoprostol. Our results also demonstrate that the quality of services and the quality of key relationships improved overall as perceived by the interviewees. We expect that if these perceived direct and indirect changes occurred, maternal outcomes will improve as a result of more women coming in contact with ANC and safer delivery services and improvements in the quality and acceptability of the services provided. This intervention has great potential for reducing maternal mortality, especially in countries with high proportions of home deliveries, where TBAs are helpless in the face of PPH. Unless trained, community-based birth attendants come to women's doorsteps, the women suffering the highest burden of maternal mortality will continue to be unreached by “available” facility-based interventions. Integrating the existing TBAs into the formal healthcare system can provide community-based points of intervention for the millions of women who will continue to deliver at home for years, if not decades, to come.

## ACKNOWLEDGEMENTS

We would like to thank Dr. Salima Rahman for her extensive help in facilitating and overseeing the project in her department at RDRS. Her cooperation was invaluable. We would also like to thank all RDRS's TBAs, CHWs, and managers who participated in this project as well as the women and the community members they serve.
